# Mechanistic Investigation of GHS-R Mediated Glucose-Stimulated Insulin Secretion in Pancreatic Islets

**DOI:** 10.3390/biom12030407

**Published:** 2022-03-06

**Authors:** Geetali Pradhan, Jong Han Lee, Chia-Shan Wu, Hongying Wang, Ligen Lin, Taraka Donti, Brett H. Graham, Arun S. Rajan, Ashok Balasubramanyam, Susan L. Samson, Shaodong Guo, Yuxiang Sun

**Affiliations:** 1 USDA/ARS Children’s Nutrition Research Center, Department of Pediatrics, Baylor College of Medicine, Houston, TX 77030, USA; gpradhan@purdue.edu (G.P.); jhleecw@gmail.com (J.H.L.); ligenl@um.edu.mo (L.L.); 2 Interdepartmental Program in Translational Biology and Molecular Medicine, Baylor College of Medicine, Houston, TX 77030, USA; 3 Department of Marine Bioindustry, Hanseo University, Seosan 31962, Korea; 4 Department of Nutrition, Texas A&M University, College Station, TX 77843, USA; chiashan.wu@ag.tamu.edu (C.-S.W.); hongying.wang@ag.tamu.edu (H.W.); shaodong.guo@tamu.edu (S.G.); 5 State Key Laboratory of Quality Research in Chinese Medicine, Institute of Chinese Medical Sciences, University of Macau, Macau 999078, China; 6 Department of Molecular and Human Genetics, Baylor College of Medicine, Houston, TX 77030, USA; taraka.donti@perkinelmer.com (T.D.); bregraha@iu.edu (B.H.G.); 7 PerkinElmer Genomics, 250 Industry Dr., Pittsburgh, PA 15275, USA; 8 Department of Medical & Molecular Genetics, Indiana University School of Medicine, Indianapolis, IN 46202, USA; 9 Department of Medicine, Baylor College of Medicine, Houston, TX 77030, USA; arajan@bcm.edu (A.S.R.); ashokb@bcm.edu (A.B.); samson.susan@mayo.edu (S.L.S.)

**Keywords:** growth hormone secretagogue receptor (GHS-R), glucose-stimulated insulin secretion (GSIS), glucose transporter 2 (*Glut2*), pancreatic and duodenal homeobox 1 (Pdx1), insulin, islets

## Abstract

Ghrelin receptor, a growth hormone secretagogue receptor (GHS-R), is expressed in the pancreas. Emerging evidence indicates that GHS-R is involved in the regulation of glucose-stimulated insulin secretion (GSIS), but the mechanism by which GHS-R regulates GSIS in the pancreas is unclear. In this study, we investigated the role of GHS-R on GSIS in detail using global *Ghsr*^−/−^ mice (in vivo) and *Ghsr*-ablated pancreatic islets (ex vivo). GSIS was attenuated in both *Ghsr*^−/−^ mice and *Ghsr*-ablated islets, while the islet morphology was similar between WT and *Ghsr*^−/−^ mice. To elucidate the mechanism underpinning *Ghsr*-mediated GSIS, we investigated the key steps of the GSIS signaling cascade. The gene expression of glucose transporter 2 (*Glut2*) and the glucose-metabolic intermediate—glucose-6-phosphate (G6P) were reduced in *Ghsr*-ablated islets, supporting decreased glucose uptake. There was no difference in mitochondrial DNA content in the islets of WT and *Ghsr*^−/−^ mice, but the ATP/ADP ratio in *Ghsr*^−/−^ islets was significantly lower than that of WT islets. Moreover, the expression of pancreatic and duodenal homeobox 1 (Pdx1), as well as insulin signaling genes of insulin receptor (IR) and insulin receptor substrates 1 and 2 (IRS1/IRS2), was downregulated in *Ghsr*^−/−^ islets. Akt is the key mediator of the insulin signaling cascade. Concurrently, Akt phosphorylation was reduced in the pancreas of *Ghsr*^−/−^ mice under both insulin-stimulated and homeostatic conditions. These findings demonstrate that GHS-R ablation affects key components of the insulin signaling pathway in the pancreas, suggesting the existence of a cross-talk between GHS-R and the insulin signaling pathway in pancreatic islets, and GHS-R likely regulates GSIS via the Akt-Pdx1-GLUT2 pathway.

## 1. Introduction

Glucose-stimulated insulin secretion (GSIS) is a tightly regulated process, and GSIS impairment is a hallmark of type 2 diabetes (T2D). Ghrelin is a peptide hormone secreted mainly from the oxyntic cells of the stomach [1,2]; it is also expressed in other cells, including the α, β, and ε-cells in pancreatic islets [3,4,5,6,7]. Further, ghrelin expression in the stomach can be regulated by both cholinergic and adrenergic arms of the autonomic nervous system [8]. Ghrelin has been reported to inhibit GSIS in the pancreas, isolated pancreatic islets, and β-cells [6,9,10,11,12]. Growth hormone secretagogue receptor (GHS-R) is the only recognized receptor for ghrelin to date [2]. GHS-R is primarily expressed in the brain and pituitary gland where it mediates ghrelin’s effects on food intake and growth hormone secretion [13]. GHS-R is also expressed in the α, β, γ, and δ-cells of pancreatic islets [13,14,15,16,17,18,19]. GHS-R mediates ghrelin-induced inhibition of insulin secretion [20], as well as the induction of glucagon [21] and somatostatin [22] in the pancreas. Various pathways have been proposed to mediate the inhibition of insulin secretion by the ghrelin—GHS-R signaling. Dezaki et al. demonstrated that ghrelin uses Gα_i/o_ to activate voltage-dependent K^+^ channels to inhibit [Ca^2+^]_I_ and insulin release [23]. Roy et al. showed that GHS-R activation leads to its coupling to Gα_i/o_ but requires heterodimerization with somatostatin receptor-5 (SSTR5) to transduce ghrelin’s inhibitory tone on insulin secretion [24]. Another study suggested that ghrelin interacts with GHS-R in β-cells to attenuate glucose-induced [Ca^2+^]_i_ and insulin release via the cAMP-TRPM2 pathway [25]. Interestingly, our studies in *ghrelin*^−/−^ and *Ghsr*^−/−^ mice showed that the ablation of *ghrelin* and *GHS-R* have distinctive effects on insulin secretion [26,27,28], which raises the question of whether ghrelin and GHS-R activate the differential signaling pathways in pancreatic β cells.

It has been widely debated whether insulin itself affects β cell function and if β cell insulin secretion is insulin-dependent. Studies using animal models, isolated islets, and cell lines have suggested that the pancreas is insulin-responsive, and insulin signaling pathways modulate β-cell functions [29]. It has been reported that insulin stimulates GSIS in the β-cells of humans [30] and rodents [31]. It has also been shown that only healthy individuals [32], not glucose tolerant/insulin-resistant individuals [33,34], are responsive to insulin in GSIS tests. Remarkably, animal data further revealed that β-cell specific insulin receptor knockout (βIRKO) mice exhibit reduced GSIS [35]; the deletion of insulin signaling proteins, such as IRS-1, IRS-2, Phosphoinositide 3-kinases (PI3K), and Protein kinase B (Akt) 2, affects insulin secretion and glucose sensing [36,37,38,39]. Glucose transporter 2 (GLUT2) is a key protein for glucose sensing in β-cells, and *Glut2* knockout mice exhibit hyperglycemia, impaired glucose tolerance, and impaired GSIS [40]. Despite these exciting findings, it is still not fully understood whether insulin signaling regulates GSIS in β-cells. Intriguingly, as nutrient-sensing regulators, both ghrelin and GHS-R have been reported to be associated with insulin resistance [27,41,42,43,44]. GHS-R has been shown to modulate the IRS1-PI3K-Akt signaling pathway in 3T3-L1 preadipocytes [45,46] and hepatoma cells [47]. However, it is unknown whether GHS-R has a similar effect on GSIS as ghrelin and whether insulin signaling mediates GHS-R-associated GSIS regulation in pancreatic islets by modulating insulin signaling.

In the current study, we used global *Ghsr*^−/−^ mice and the *Ghsr*-ablated pancreatic islets to investigate the molecular mechanisms by which GHS-R regulates GSIS in pancreatic islets.

## 2. Materials and Methods

### 2.1. Animals

Animals were housed under controlled temperature and lighting cycles (75 ± 1 °F; 12 h light–dark cycle) with free access to regular mouse chow and water. *Ghsr*^−/−^ mice in a C57BL/6J background were generated and characterized as we previously described, which were backcrossed to a C57BL/6J background for 15 generations [13]. All mice used in the experiments were age-matched congenic males unless otherwise indicated. We only used male mice in our study to eliminate insulin variation associated with menstrual cycles of female mice [48]. All mice were housed and bred in a pathogen-free facility at Baylor College of Medicine, and the animal protocol was approved by the Animal Care Research Committee at the Baylor College of Medicine.

### 2.2. Glucose Stimulated Insulin Secretion (GSIS) In Vivo

Mice were fasted overnight (16 h) and then intraperitoneally (i.p.) injected with 3 g/kg body weight glucose. Blood glucose was measured at 0, 2, 5, 15, and 30 min, and insulin was measured at 0, 2, 5, 15, and 30 min after glucose administration. Glucose was measured using OneTouch Ultra2 glucometer (LifeScan Inc., Milpitas, CA, USA). Insulin was measured using Mouse Insulin ELISA kit (Mercodia, Uppsala, Sweden) per the manufacturer’s instructions.

### 2.3. Immunohistochemistry of Pancreas

Immunostaining was performed on pancreatic sections fixed in formalin and paraffin-embedded by the Texas Medical Center Digestive Diseases Center, Cellular and Molecular Morphology core. The glucagon antibody (Cat# PA0594) and insulin antibody (2D11-H5, Cat# Pa0620) were obtained from Leica Biosystems (Wetzlar, Germany). The image in the manuscript is representative of 12–14 islets/mouse, 3 mice/genotype.

### 2.4. Islet Isolation

The islets were collected using the collagenase method, digested with collagenase P (Roche, Basel, Switzerland) and then were cultured overnight, as we and others have described [49,50]. Briefly, 3 mL of 1 mg/mL collagenase P was injected with a 27G needle into the ampulla of Vater of the pancreas. The digested pancreas was then removed and placed in a 50 mL tube containing 2 mL of the collagenase solution. The tube was subsequently placed in 37 °C shaking water bath shaking at 100–120 rpm speed for 12–13 min until the solution became homogenous. Cold Hank’s Balanced Salt Solution (20 mL) was later added to stop digestion. The tubes were centrifuged at 290× *g* for 30 s at 4 °C, and the supernatant decanted. This procedure was repeated 3 more times; the islets were then separated from the acinar tissue using a Ficoll gradient Histopaque-1077 (Sigma-Aldrich, Kawasaki, Japan) per the manufacturer’s instructions. The islets were subsequently hand-picked under a microscope and incubated overnight in RPMI 1640 medium containing 5.5 mM glucose at 37 °C. The next day, the medium was replaced with Hanks’ Balanced Salt solution (HBSS) pH 7.2, consisting of 114 mM NaCl, 4.7 mM KCl, 1.2 mM KH_2_PO_4_, 1.16 mM MgSO_4_, 20 mM HEPES, 2.5 mM CaCl_2_, 25.5 mM NaHCO_3_^,^ 0.2% bovine serum albumin, and 3.3 mM glucose, and incubated for 2 h at 37 °C before assessing GSIS ex vivo [51].

### 2.5. Glucose-Stimulated Insulin Secretion (GSIS)

After overnight recovery in RPMI (5.5 mM glucose), the islets were placed in HBSS solution containing 3.3 mM glucose for 2 h. Then, 10 similar-sized islets were hand-picked and placed in each well of a 24-well plate. There were 8–12 wells per treatment/genotype in each experiment. The islets were incubated in 500 µL HBSS containing 3.3 mM, 11.1 mM, or 22.2 mM glucose, and the plate was placed in a 37 °C incubator for 2 h. After 2 h, the supernatant was saved for insulin measurement, and the islets in each well were picked to measure protein content. The insulin secreted in the supernatant was normalized to the protein content in islets.

### 2.6. Insulin Content of Pancreas and Islets

For the total insulin content of the pancreas, the whole tissue was weighed and then homogenized in 3 mL of ice-cold acid-ethanol, containing 70% (*v/v*) ethanol and 5% (*v/v*) HCl. The tubes were then held at 4 °C for 24 h. After 24 h, 2 mL ice-cold acid-ethanol was added to the tubes, tissue was homogenized, and tubes were held at 4 °C for another 24 h. On day 3, tubes were centrifuged at 2400 rpm for 30 min at 4 °C, and the supernatant was saved for insulin measurement. The insulin content was normalized to the weight of the pancreas. For the insulin content of islets, 15 pancreatic islets were homogenized using 100 µL of ice-cold acid-ethanol. A total of 10 µL of the lysate was used to measure the protein content using a Bradford assay. The remaining lysate was held at 4 °C for 24 h. After 24 h, the tubes were centrifuged at 12,000 rpm for 15 min at 4 °C, and the supernatant was saved for insulin measurement. The insulin content in the islets was normalized to the protein content and the number of islets.

### 2.7. Respirometry of Islets

The Seahorse XF24 Extracellular Flux Analyzer (Seahorse Bioscience, Copenhagen, Denmark) was used to assess a range of metabolic parameters through real-time monitoring of the cellular Oxygen Consumption Rate (OCR). Islets were isolated and incubated overnight in RPMI supplemented with 5.5 mM glucose. On the day of the assay, the islets were incubated in HBSS buffer containing either 3.3 mM glucose or 22.2 mM glucose for 2 h. Then, the islets were washed in a 50 mL tube containing Seahorse Assay media + 3.3 mM glucose. Then, the media was carefully removed from the 50 mL tube, and the islets were resuspended in another 2 mL of Seahorse Assay media + 3.3 mM glucose. A total of 50–80 islets were seeded/well in XF 24-well islet capture microplates by pipetting 2 × 50 µL of stirred islet mix into each of the 20 wells, pre-loaded with 400 mL of Seahorse Assay media + 3.3 mM glucose. Four wells were kept empty as controls in every experiment. Screens were carefully put on top of the depression of all wells with tweezers. To avoid bubble formation, the screens were pre-wetted with Seahorse Assay media. The islet plate was then incubated for 60 min at 37 °C without CO_2_, before it was loaded into the XF24 respirometry machine. Four baseline OCR measurements were taken before the following substrates/compounds were injected: 22.2 mM glucose, 5 μM oligomycin, 1 μM 2-[2-[4-(trifluoromethoxy)phenyl]hydrazinylidene]-propanedinitrile (FCCP), 5 μM rotenone, and 5 μM antimycin A. A change in OCR after 22.2 mM glucose treatment is obtained by subtracting OCR after 22.2 mM glucose treatment from the basal OCR value. The rate of oxygen consumption coupling to ATP production (ATP turnover) is obtained by subtracting the Oligomycin-associated OCR value from basal OCR. The data are normalized to the protein content of islets/well and are presented as pmol/min/mg protein.

### 2.8. ATP/ADP Ratio

On the day of the assay, the islets were placed in HBSS containing 1.1 mM glucose for 2 h. Then, 15 similar-sized islets/well were hand-picked and treated with 3.3 mM glucose or 22.2 mM glucose. After treatment for 2 h, the ATP/ADP ratio was measured using ApoSENSOR ADP/ATP Ratio Bioluminescent Assay Kit (BioVision, Milpitas, CA, USA), per the manufacturer’s instructions.

### 2.9. Glucose-6-Phosphate Measurement

The animals were fasted overnight for 18 h, then injected intraperitoneally with 3 g/kg glucose. After 1 h, the pancreas was removed, and Glucose-6-Phosphate (G6P) was measured using Glucose-6-Phosphate Assay Kit (Sigma-Aldrich, St. Louis, MO, USA), per the manufacturer’s instructions.

### 2.10. Real-Time RT-PCR

Total RNA from islets was isolated using Arcturus PicoPure RNA Isolation Kit, Catalog # KIT0202, KIT0204 (ABI), following the manufacturer’s instructions. The cDNA was synthesized from 250–500 ng RNA using the SuperScript III First-Strand Synthesis System (Invitrogen, Carlsbad, CA, USA). Real-time RT-PCR was performed on Bio-Rad Real-Time PCR Cycler (Bio-Rad Lab., Hercules, CA, USA) using SYBR Green PCR Master Mix according to the protocol provided by the manufacturer. Relative gene expression levels were normalized by 18S rRNA and/or β-actin. The primers were as follows, *Glut2*: forward primer 5′-ATCATTGGCACATCCTACT-3′, reverse primer 5′-TCAGTTCCTCTTAGTCTCTTC-3′; IR: forward primer 5′-CAAAAGCACAATCAGAGTGAGTATGAC-3′, reverse primer 5′-ACCACGTTGTGCAGGTAATCC-3′; IRS-1: forward primer 5′-GCCTGGAGTATTATGAGAACGAGAA-3′, reverse primer 5′-GGGGATCGAGCGTTTGG-3′; IRS-2: forward primer 5′-acttcccagggtcccactgctg-3′, reverse primer 5′-ggctttggaggtgccacgatag-3′; Pdx1: forward primer 5′-AGAGGGGGAACGACTCTAGG-3′, reverse primer 5′-ACTTGAGCGTTCCAATACGG-3′; MafA: forward primer 5′-CGCAGGCCACCACGTGCGCTTGGAGGAG-3′, reverse primer 5′-CTGCGCTGGCGAGGGCTCCCGAGGGAAG-3′. Ins 1: forward primer 5′-GACCAGCTATAATCAGAGACC-3′, reverse primer 5′-AGTTGCAGTAGTTCTCCAGCTG-3′; Ins 2: forward primer 5′-AGCCCTAAGTGATCCGCTACAA-3′, reverse primer 5′-AGTTGCAGTAG-TTCTCCAGCTG-3′.

### 2.11. Western Blot

Protein expression of PDX1 was measured from the pancreas of 5-month-old WT and *Ghsr*^−/−^ mice. To measure the effects of insulin on the phosphorylation of Akt, WT, and *Ghsr*^−/−^, mice were fasted for 24 h. Then, the mice were injected with 10U insulin or saline ip. After 5 min, pancreatic tissue was collected for assessing protein expression of p-AKT and t-AKT [52]. Antibodies for p-Akt, t-Akt, Pdx1, and β actin were obtained from Cell Signaling Technologies (Beverly, MA, USA). For immunohistochemical staining, the following primary antibodies were used: rabbit anti-mouse p-Akt (1:1000), rabbit anti-mouse t-Akt (1:1000), rabbit anti-mouse pdx1 (1:1000), and rabbit anti-mouse β actin (1:1000). Goat anti-rabbit IgG was used as a secondary antibody at a 1:200 dilution.

### 2.12. Extraction and Quantification of Mitochondrial DNA

Mitochondrial DNA (mtDNA) was extracted and quantified as described with modification [53]. Briefly, pancreatic islets were isolated and homogenized in isolation buffer (300 mM sucrose, 1 mM EDTA, 5 mM MOPS, 5 mM KH2PO4, 0.01% BSA, pH 7.4) in a glass homogenizer with Teflon pestle. The homogenate was first filtered through a layer of gauze and centrifuged at 8000× *g* for 10 min at 4 °C. The supernatant was discarded, and the pellet (containing cell debris, nuclei, and mitochondria) was resuspended in a small amount of isolation buffer and transferred to a new tube. Then the tube was centrifuged at 800× *g* for 10 min at 4 °C, and the supernatant (containing mitochondria) was carefully transferred to a new tube, and the pellet containing the nuclei (including cell debris) was saved. The new supernatant was then further centrifuged at higher speed of 8000× *g* for 10 min at 4 °C, and the resulting mitochondria pellet was saved. The nuclear DNA was extracted with the phenol/chloroform method [54]. An aliquot of the mitochondrial fraction was digested overnight in lysis buffer (10 mM Tris, pH 8.0, 10 mM EDTA, 10 mM NaCl, 0.5% SDS, 100 mg/mL Proteinase K) at 37 °C, and then boiled for 5 min. The mitochondrial DNA was linearized by digestion with Bcl-I for 3 h at 50 °C and then boiled for 5 min. The samples were centrifuged at 7000× *g* for 5 min, and the resulting supernatant was used for subsequent PCR amplification. PCR was performed to amplify a 162-nt region of the mitochondrial NADH dehydrogenase subunit 4 gene. The primer sequences were 5′-TACACGATGAGGCAACCAAA-3′ and 5′-GGTAGGGGGTGTGTGTTGTGAG-3′. The PCR product was purified with the High Pure PCR template preparation kit (Roche, Indianapolis, IN). The nuclear DNA and the amplified PCR product of mitochondrial DNA (mtDNA) were quantified with NanoDrop (ND-1000 Thermo Scientific, Waltham, MA, USA), and the ratio of mtDNA/total DNA was calculated.

### 2.13. Statistical Analysis

Graph-Pad Prism version 6.0 software was used. Two-way ANOVA with repeated measures or one-way ANOVA was used. Data are represented as mean ± SEM, and *p* < 0.05 was considered statistically significant.

## 3. Results

### 3.1. GHS-R Ablation Decreases Glucose-Stimulated Insulin Secretion (GSIS)

**Figure 1:**
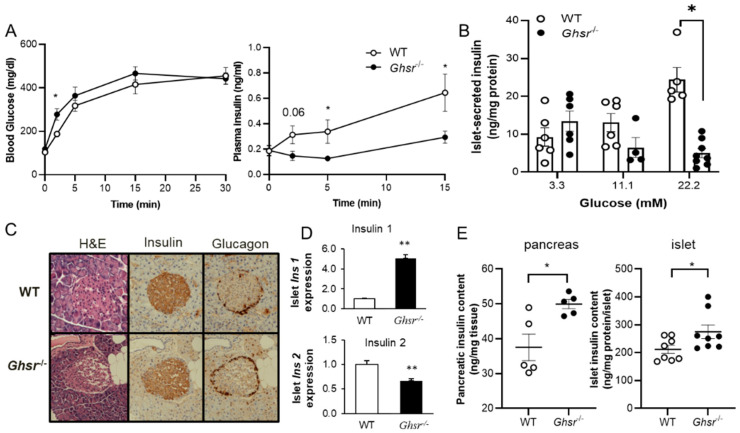
*GHS-R* ablation decreases glucose-stimulated insulin secretion (GSIS). (**A**) 4.5-month-old WT and *Ghsr*^−/−^ mice were fasted overnight, and GSIS was performed in vivo, *n* = 5. (**B**) Islets from 4-month-old WT and *Ghsr*^−/−^ mice were treated with 3.3, 11.1 or 22. 2 mM glucose for 2 h. Insulin content was measured and normalized to the total protein content of islets, *n* = 5–8. (**C**) H&E staining and immunostaining of insulin and glucagon of pancreatic sections from WT and *Ghsr*^−/−^ mice. (**D**) *Ins1* and *Ins2* gene expression was measured from islets of 6.5-month-old mice, *n* = 5. (E) Insulin protein content of the whole pancreas (4 months, *n* = 5) and islet (5.5 m, *n* = 8) was measured and normalized by total protein content. * *p* < 0.05, ** *p* < 0.001, WT vs. *Ghsr*^−/−^.

To access the effect of *Ghsr* ablation on GSIS, we performed a GSIS assay in vivo using wild-type (WT) controls and *Ghsr*^−/−^ mice. Insulin secretion is biphasic, traditionally present as first and second phases. A regular glucose tolerance test (2.0 g/kg glucose for 2 h) cannot easily distinguish between the two phases of insulin secretion patterns, so we performed a 30 min GSIS test using 3.0 g/kg glucose. The blood glucose was mostly similar between WT controls and *Ghsr*^−/−^ mice in the GSIS assay, except at the 2 min time point where glucose was significantly higher in *Ghsr*^−/−^ mice (Figure 1A). Interestingly, insulin secretion in *Ghsr*^−/−^ mice was significantly attenuated during GSIS, especially at the 5 and 15-min points (Figure 1A), which indicates that first-phase insulin secretion was decreased. We have previously reported lower fasting insulin in 6-month-old *Ghsr*^−/−^ mice [55]; interestingly, the 4.5-month-old *Ghsr*^−/−^ mice showed similar fasting insulin at the time point of in vivo GSIS (Figure 1A). To directly assess the effect of *Ghsr* deletion on GSIS, pancreatic islets from WT and *Ghsr*^−/−^ mice were treated with 3.3, 11.1, or 22.2 mM glucose, and insulin levels were measured. While insulin secretion at the lowest glucose concentration (3.3 mM) was similar between the two genotypes, the insulin secreted by *Ghsr*^−/−^ islets was significantly reduced at higher glucose concentrations of 11.1 and 22.2 mM (Figure 1B). To determine whether reduced insulin detected during GSIS was due to the low availability of insulin stored in β-cells, the islet morphology and total insulin content in the entire pancreas and isolated islets of WT and *Ghsr*^−/−^ mice were assessed. Grossly, no obvious difference was observed in islet size or intensity of glucagon staining in the pancreatic sections, while the insulin staining appeared to be denser in *Ghsr*^−/−^ mice (Figure 1C). Rodents have two insulin genes, namely Insulin 1 and Insulin 2 [56,57], which are normally expressed in a ratio of 1:2 simultaneously [58]. To determine insulin gene expression, we assessed Insulin 1 (Ins1) and Insulin 2 (Ins2) in pancreatic islets. Intriguingly, the islet *Ins1* gene expression was significantly upregulated, whereas *Ins2* gene expression was significantly downregulated in *Ghsr*^−/−^ mice (Figure 1D). The total insulin protein content in the pancreas and islets of *Ghsr*^−/−^ mice was higher compared to WT controls (Figure 1E). Overall, these results indicate that *GHS-R* ablation suppresses GSIS both in vivo and ex vivo, despite the higher insulin content in the islets, which suggests that GHS-R affects insulin secretion but not insulin production.

### 3.2. Ghsr^−/−^ Islets Exhibit Lower ATP/ADP Ratio

**Figure 2:**
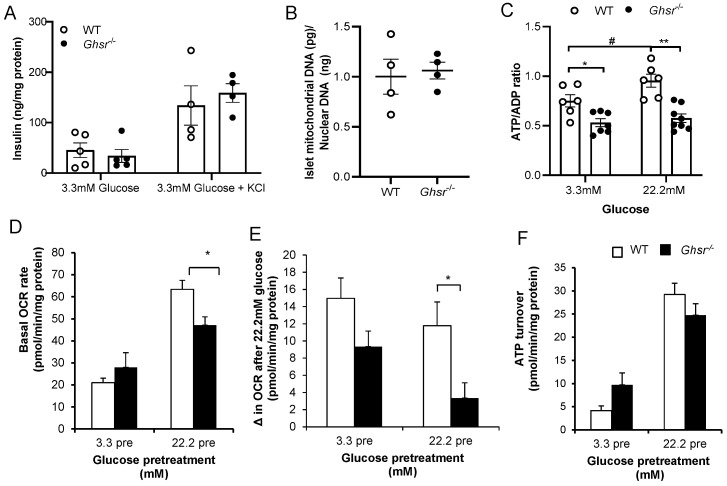
*Ghsr*^−/−^ islets have a lower ATP/ADP ratio. (**A**) Islets from WT and *Ghsr*^−/−^ mice were treated with 3.3 mM glucose in the presence or absence of 30 mM KCl, and insulin was measured after 2 h. Insulin was normalized to total protein content in islets, *n* = 4–5. (**B**) Ratio of mitochondrial DNA to nuclear DNA content in islets. *n* = 4. (**C**) 15 Islets/well from WT and *Ghsr*^−/−^ mice were treated with 3.3 or 22.2 mM glucose for 2 h, and then the ATP/ADP ratio was measured, *n* = 6–8. (**D**) OCR was assessed in islets of WT and Ghsr^−/−^ mice. Islets of WT and Ghsr^−/−^ mice were pretreated with 3.3 or 22.2 mM glucose for 2 h, and then the basal OCR rate was measured. (**E**) Change in OCR from baseline after 22.2 mM glucose treatment. (**F**) ATP turnover, *n* = 3–5. * *p* < 0.05, WT vs. *Ghsr* ^−/−^; ** *p* < 0.001, WT vs. *Ghsr*^−/−^; # *p* < 0.05, 3.3 mM glucose vs. 22.2 mM glucose (WT).

To elucidate the mechanism underpinning *Ghsr*-deficiency-induced GSIS impairment, we investigated all the key steps of the GSIS signaling cascade as outlined below [59]. Glucose stimulation is known to induce the translocation of glucose transporter GLUT2 to the cell membrane, where it promotes the uptake of glucose molecules into the cytosol. The glucose is then hydrolyzed to generate ATP in the mitochondria. The increase of ATP leads to the closure of ATP-sensitive K+ ATP channels in the cell membrane, which leads to depolarization of the membrane. This change in membrane voltage leads to the opening of voltage-sensitive Ca2+ channels and an influx of Ca2+ ions into the cytosol, which ultimately results in the exocytosis of the insulin granules [59]. To determine if GHS-R affects the membrane depolarization step, we treated the islets with KCl, which is known to directly depolarize the membrane [60,61]. As detailed in Figure 2A, the insulin secreted from both WT and *Ghsr*^−/−^ islets were similar after the KCl treatment, which suggests that *Ghsr* ablation likely impairs the upstream membrane depolarization step of the insulin secretion signaling cascade. Altered mitochondrial function is known to impair GSIS [62]. The DNA content in mitochondria was similar between the islets of the WT and *Ghsr*^−/−^ mice (Figure 2B). To test mitochondrial function, we further investigated the ATP/ADP ratio in islets of WT and *Ghsr*^−/−^ mice. As shown in Figure 2C, the ATP/ADP ratio in WT islets was increased in 22.2 mM glucose treatment compared to that of 3.3 mM glucose treatment; the ATP/ADP ratio in *Ghsr*^−/−^ islets was comparable under both 3.3 and 22.2 mM glucose concentrations. Importantly, the ATP/ADP ratio in *Ghsr*^−/−^ islets was significantly decreased at both 3.3 and 22.2 mM glucose concentrations compared to WT islets (Figure 2C). These data indicate that *Ghsr* deletion does not impact the number of mitochondria but does impair the function of mitochondria.

To determine why mitochondrial function was altered, we further assessed the OCR of islets pretreated with either low glucose (3.3 mM) or high glucose (22.2 mM). As expected, islets treated with low glucose had a much lower basal OCR compared to islets treated with high glucose. The basal OCR of the *Ghsr*^−/−^ islets treated with high glucose for 2 h was significantly lower than the WT islets treated with high glucose (Figure 2D). Upon further stimulation by glucose (22.2 mM), we observed strikingly reduced OCR in *Ghsr*^−/−^ islets pretreated with high glucose (Figure 2E). ATP turnover showed a trend of decrease in the *Ghsr*^−/−^ islets pretreated with 22.2 mM glucose but failed to reach statistical significance (Figure 2F). Together these findings suggest lower availability of substrates in the mitochondria of *Ghsr*^−/−^ islets, which may contribute to reduced ATP generation.

### 3.3. Glucose Uptake Is Reduced in Ghsr-Ablated Islets

**Figure 3:**
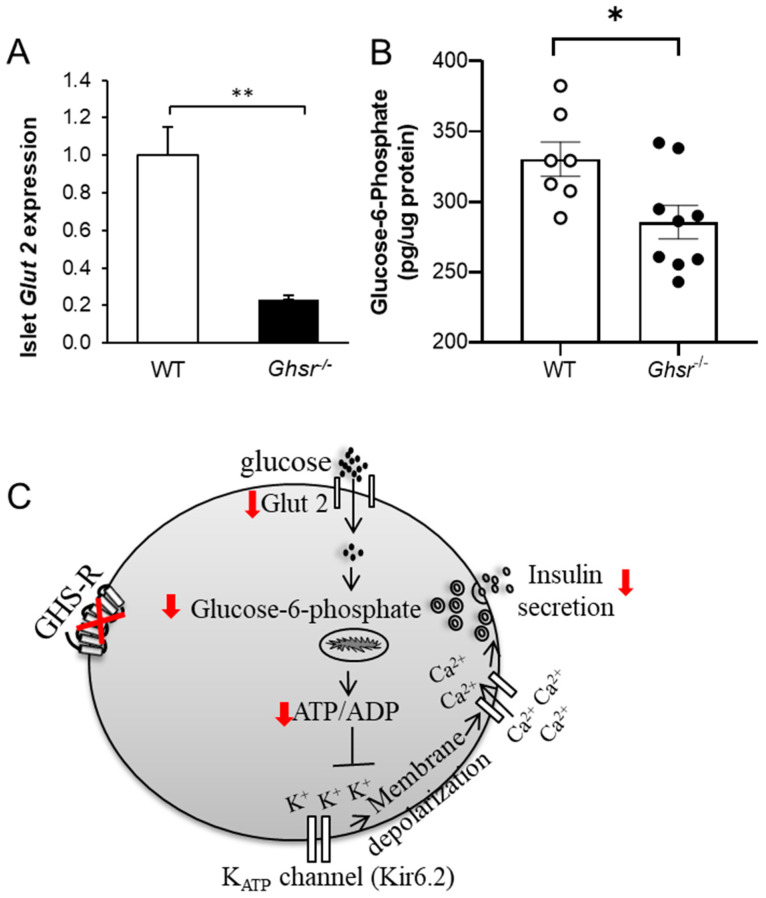
Glucose uptake is reduced in *Ghsr*^−/−^ mice. (**A**) *Glut2* gene expression was measured in islets of 6.5-month-old WT and *Ghsr*^−/−^ mice, *n* = 5. (**B**) G6P was measured from the pancreas of 4-month-old WT and *Ghsr*^−/−^ mice, which were fasted overnight, injected with glucose and then sacrificed 60 min later, *n* = 7–9. (**C**) Schematic diagram of impairments in GSIS pathway in *Ghsr*-ablated β-cells. Impaired *Glut2* expression leads to the reduced formation of G6P, contributing to the lower generation of ATP, resulting in attenuated insulin secretion. * *p* < 0.05, ** *p* < 0.001, WT vs. *Ghsr*^−/−^.

GLUT2 is the primary glucose transporter in rodent β-cells and plays a crucial role in glucose sensing and uptake [63]. The gene expression of *Glut2* was significantly reduced in islets of *Ghsr*^−/−^ mice (Figure 3A). G6P is known as the first substrate produced after glucose is transported into the β-cells [64]. Consistent with reduced *Glut2* expression, we detected reduced G6P content in the pancreas of *Ghsr*^−/−^ mice (Figure 3B). Our results indicate impaired GSIS in *Ghsr*^−/−^ islets is likely due to reduced uptake of glucose into the cytosol, which produces lower G6P levels, subsequently leading to a lower ATP/ADP ratio. The reduced ATP/ADP ratio then triggers insufficient membrane depolarization, which subsequently results in reduced GSIS (Figure 3C).

### 3.4. Identification of Molecular Mediators Governing GHS-R Induced GSIS

**Figure 4:**
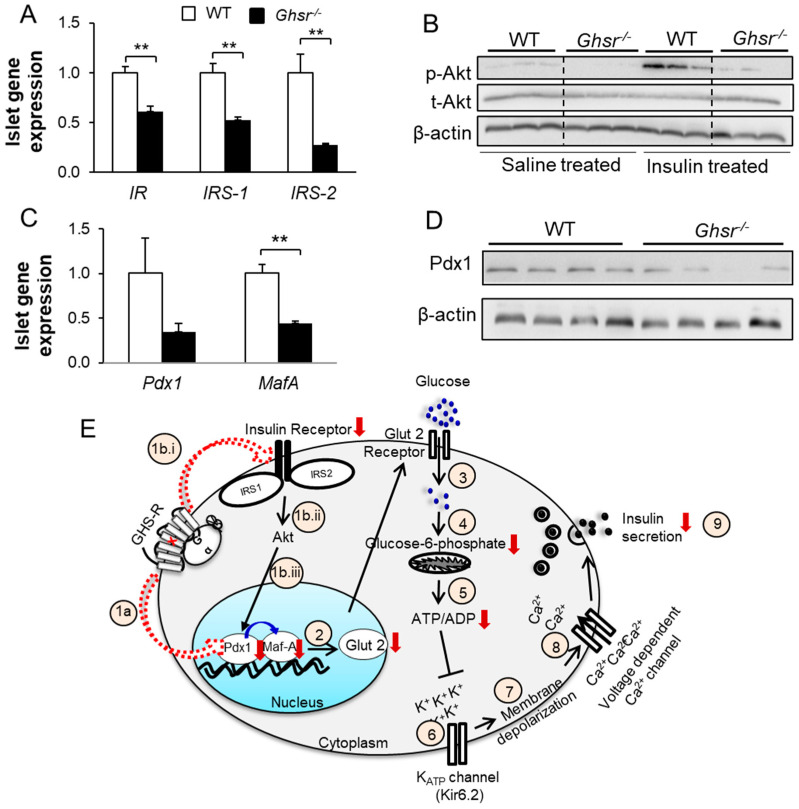
Genes of IR-IRS1/IRS2 pathway and transcription factor Pdx1 are downregulated in *Ghsr*^−/−^ mice. (**A**) Gene expression of IR, IRS1, and IRS2 were measured in islets of 6.5-month-old mice, *n* = 5. (**B**) Protein expression of p-AKT and t-AKT was measured in the pancreas from 12–14-month-old WT and *Ghsr*^−/−^ mice injected with saline or insulin. (**C**) Gene expression of transcription factors, *Pdx1*, and *MafA* were measured in islets of 6.5-month-old mice, *n* = 5. (**D**) Protein expression of Pdx1 was measured in the pancreas of 5-month-old WT and *Ghsr*^−/−^ mice, *n* = 4. (**E**) Schematic diagram illustrating that GHS-R regulates GSIS via the AKt-Pdx1 pathway. The numbers in the diagrams are denoted as below: (1) GHS-R ablation in β-cells downregulates Pdx1 and MafA either directly or indirectly reducing the expression of insulin signaling of IR, IRS1, IRS2, and Akt; (2) The downregulation of Pdx1 and MafA impairs *Glut2* expression; (3) Reduced *Glut2* expression leads to decreased glucose uptake; (4) Reduced generation of Glucose-6-phosphate leads to lower ATP/ADP ratio; (5) Reduced ATP/ADP ratio causes partial closing of ATP sensitive K_ATP_ channels (6), which leads to insufficient membrane depolarization (7); This ultimately results in a reduced influx of Ca^2+^ ions (8) that suppresses insulin secretion (9). ** *p* < 0.001, WT vs. *Ghsr*^−/−^.

Components of the insulin signaling pathway have been previously suggested to regulate GSIS in pancreatic islets [35,39]. To investigate whether the insulin signaling pathway is altered in our system, the levels of gene expression of IR, IRS1, and IRS2 were measured. The expressions of *IR*, *IRS1*, and *IRS2* were significantly downregulated in *Ghsr*^−/−^ islets mice compared to WT islets (Figure 4A). Next, we activated the insulin signaling pathway by injecting the mice with insulin and measured the phosphorylation of Akt. Under basal conditions (saline treatment), the phosphorylation of Akt seemed to be less in the pancreas of *Ghsr*^−/−^ mice compared to WT. After insulin treatment, the phosphorylated Akt was dramatically increased in the WT pancreas as expected, and the increase in the *Ghsr*^−/−^ pancreas was much less pronounced (Figure 4B).

A recent report demonstrated that the knockdown of IR in pancreatic β-cells contributes to reduced GSIS by downregulating the Pdx1-GLUT2 pathway [65]. Next, we set to determine if the downregulation of the IR-IRS1/IRS2-PI3K/Akt signaling pathway observed in *Ghsr*^−/−^ islets is linked to reduced expression of Pdx1. Pdx1 and MafA are important transcription regulators in β-cells that are well known to regulate the expression of GLUT2 [66,67,68,69]. We observed lower expression of both *Pdx1* and *MafA* in *Ghsr*^−/−^ islets, though the difference in *Pdx1* expression was not significant (Figure 4C). Consistently, the protein expression of Pdx1 was attenuated in the pancreas of *Ghsr*^−/−^ mice compared to WT mice (Figure 4D). Overall, GHS-R ablation in β-cells downregulates Pdx1 and MafA, and suppresses the expression of key components of the IR-IRS1/IRS2-Akt pathway. It is possible that the downregulation of Pdx1 impairs *Glut2* expression to reduce glucose uptake, which in turn suppresses G6P generation. Reduced G6P leads to a reduced ATP/ADP ratio, which then causes partial closure of K_ATP_ channels and insufficient membrane depolarization, subsequently resulting in the reduced influx of Ca^2+^ ions and attenuated GSIS (Figure 4E).

## 4. Discussion

This is a comprehensive analysis of the GHS-R signaling on GSIS in *Ghsr*^−/−^ mice and islets. Our findings suggest that GHS-R regulates GSIS via the Akt-Pdx1-GLUT2 pathway. We demonstrated that there is downregulation of GSIS in young *Ghsr*^−/−^ mice and in isolated pancreatic islets. Our data showed that the downregulation of insulin secretion is likely due to reduced glucose transport into islets, supported by reduced *Glut2* expression and decreased G6P levels, which results in low ATP generation. Due to the reduced ATP/ADP ratio, the downstream depolarization of the membrane via K^+^_ATP_ channels and subsequent Ca^2+^ influx is abrogated, leading to decreased insulin secretion. The reduced *Glut2* expression observed in *Ghsr*^−/−^ islets could be attributed to the reduced expression of Pdx1 in these mice. Interestingly, we also detected lower gene expression of key insulin signaling genes such as IR, IRS1 and IRS2, as well as lower expression of phosphorylated Akt. Moreover, the insulin-stimulated phosphorylation of Akt was reduced in the pancreas of *Ghsr*^−/−^ mice, further supporting the involvement of insulin signaling. Together, these results suggest that GHS-R regulates glucose-induced insulin release by regulating the glucose uptake via the Akt-Pdx1-GLUT2 pathway, supporting the existence of a crosstalk between the GHS-R and the insulin signaling pathway in the pancreatic islets.

In the pancreas, GHS-R mediates ghrelin’s effects on insulin, glucagon, and somatostatin secretion [20,21,22]. Previously, we have extensively studied GHS-R null mouse models and demonstrated that young *Ghsr*^−/−^ mice had similar body weight, body composition, food intake, and energy expenditure as control mice [53]. We also reported that ghrelin -GHS-R signaling plays an important role in regulating glucose homeostasis [44,70,71]. Our *Ghsr*^−/−^ mice exhibited reduced glucose and insulin after overnight fasting [55], similar to the observation of Zigman et al. in their *Ghsr*-null mice [72]. In the present study, we found reduced GSIS in *Ghsr*-ablated islets and reduced first-phase insulin secretion in the *Ghsr*^−/−^ mice compared to WT controls, consistent with our report of *Ghsr*- and *leptin*-deleted *Ghsr*^−/−^*:ob/ob* mice [28]. In our β-cell-specific GHS-R-deleted mouse model, where *Ghsr* was deleted only in β-cells, the first-phase insulin secretion was reduced in vivo and ex vivo accompanied with improved insulin sensitivity, but no changes in body weight, body composition, food intake, or energy expenditure [73]. Together, all our data are in support of β-cell GHS-R as an important regulator of GSIS. However, our results differ from the study by Gray et al., where they reported similar GSIS in *Ghsr*-null mice and their islets [74]. We used 4.5–6.5 month-old mice/islets, Gray et al. used 2–2.5 month-old mice and 2–3-month-old islets. Mice undergo dramatic developmental changes between 2 and 6 months; the age difference may explain the discretionary of the data.

The morphology of *Ghsr*^−/−^ islets appeared similar to WT islets, indicating that reduced GSIS is likely caused by GHS-R associated functional changes, not structural changes. In *Ghsr*^−/−^ islets, the Insulin 1 gene was increased, whereas the Insulin 2 was significantly decreased (Figure 1). This increase of *Ins1* could be a compensatory response to the downregulation of *Ins2* in *Ghsr*-null islets. This is consistent with previous observation in *Ins2*-knockout islets that exhibit elevated Insulin 1 expression [75]. Similar differential expression of Insulin 1 and Insulin 2 have also been reported in β-cells under glucose-stimulated conditions, with increased Insulin 1 expression and no change in Insulin 2 expression [76]. Notably, the insulin content in the whole pancreas and isolated islets was increased in the *Ghsr*^−/−^ mice, which could be a consequence of increased Insulin 1 expression and reduced secretion of insulin, further reserving the pool of insulin in the cytosol. These results indicate that *Ghsr*^−/−^ islets have no inherent defect in their islet structure and overall total insulin production, but have the functional impairment of diminished exocytosis of insulin under glucose stimulation.

Mitochondrial metabolism plays a pivotal role in regulating GSIS; mitochondrial coupling and oxygen consumption are impaired in diabetic islets [77]. It has been previously reported that the oxygen consumption rate of β-cells is increased after glucose stimulation, suggesting accelerated β-cell metabolism leads to increased insulin secretion [78]. Our analysis of mitochondrial function in *Ghsr*^−/−^ mice showed that oxygen consumption was reduced in the islets after 2 h of exposure to hyperglycemic conditions (Figure 2D). The reduced OCR and β-cell metabolism observed in 22.2 mM glucose-pretreated *Ghsr*-ablated islets could lead to the reduced GSIS observed in these mice (Figure 1). Upon further stimulation with 22.2 mM glucose, the OCR change in *Ghsr*^−/−^ islets pretreated with 3.3 mM glucose was more than the islets pretreated with 22.2 mM glucose (Figure 2E). This is consistent with a previous study that hyperglycemia is associated with reduced mitochondrial metabolism in β-cells [77]. Mitochondrial metabolism has significant functional consequences, downstream in the GSIS pathway, as reduced ATP impairs membrane depolarization, calcium influx, and insulin exocytosis. Consequently, treatment of the *Ghsr*^−/−^ islets with a classical depolarizing agent, KCl, sufficiently depolarized the membrane to restore insulin secretion to the levels of control mice.

Human β-cells express three different glucose transporters, GLUT1, GLUT2, and GLUT3 [79], while GLUT2 is the primary glucose transporter in rodent β-cells [63]. The loss of *Glut2* expression in humans has been associated with impaired GSIS and the loss of first-phase insulin secretion in mice [80]. In the present study, *Glut2* gene expression was reduced in *Ghsr*-ablated islets. Consequently, glucose uptake in β-cells was likely attenuated, which resulted in lower phosphorylation of glucose and reduced G6P in *Ghsr*^−/−^ mice (Figure 3). Pdx1 is a key transcription regulator of β-cells, which is known to regulate expression by binding to the transcription promoter region of *Glut2* [66,67], *Insulin* [66], and *MafA* [81]. Partial downregulation of Pdx1 expression is associated with β-cell dysfunction and β-cell death in rodents [82]. The lower *Glut2* expression that we observed in *Ghsr*^−/−^ mice could be a consequence of downregulated Pdx1 expression in these mice, which is consistent with previous reports that reduced expression of Pdx1 results in the suppression of *Glut2* [67,83]. MafA is a β-cell specific transcriptional factor that has been shown to induce the expression of *Glut2* [68,69], *Insulin* [84,85], and *Pdx1* [68,86]. Interestingly, we observed a significant downregulation of MafA expression in the pancreatic islets of *Ghsr*^−/−^ mice, which could be a result of Pdx1 downregulation in these mice. Our findings suggest that the downregulation of Pdx1 and MafA in *Ghsr*^−/−^ β-cells possibly contributes to reduced Glut 2 expression, which eventually leads to the attenuation of glucose-stimulated insulin secretion.

Traditionally, insulin signaling and insulin secretion in the β-cells have been thought to be distinctive independent pathways. However, recent research has demonstrated that insulin can stimulate GSIS in humans [30] and rodents [31]. β-cell specific insulin receptor knockout (βIRKO) mice exhibit loss of first-phase insulin secretion in response to glucose [35]. Low levels of IR and IRS2 have been reported in islets of diabetic humans [87], whereas IRS2 overexpression reduces the incidence of diabetes in non-obese diabetic mice [88]. Furthermore, insulin treatment of islets increases the nuclear translocation of Pdx1 [89], and IR isoform A improves glucose uptake by its association with Glut1/2 in β-cells [90]. A recent report demonstrated that the knockdown of IR in pancreatic β-cells (INS-1) contributes to reduced GSIS mediated by downregulating the Pdx1-GLUT2 pathway [65]. It is possible that the attenuation of the Pdx1-GLUT2 pathway is a direct consequence of reduced insulin signaling in the β-cells of *Ghsr*^−/−^ mice. The activation of GHS-R has been shown to modulate IRS1-associated PI3K/Akt signaling in various cell types in vitro. In 3T3-L1 preadipocytes it has been reported that ghrelin via GHS-R activates the IRS1-PI3K-Akt signaling pathway to induce proliferation and insulin-stimulated glucose uptake [45,46]. In hepatoma cells, GHS-R activation ameliorates IRS1-associated PI3K activity and suppresses Akt activity [47]. Here we are investigating whether GHS-R crosstalk with the IR-IRS1/IRS2-Akt pathway in the pancreatic β-cells. Activation of the insulin receptor upon ligand binding triggers a series of phosphorylation events, including phosphorylation of IRS1 and IRS2. The IRS proteins then activate the PI3K-Akt pathway, where Akt is phosphorylated [91]. Consistently, we observed an increase in insulin-induced Akt phosphorylation in the pancreas of WT mice, whereas Akt phosphorylation was reduced in the pancreas of *Ghsr*^−/−^ mice (Figure 4). The evidence of reduced gene expression and signaling of IR-IRS1/IRS2-Akt pathway in the pancreas of *Ghsr*^−/−^ mice suggests crosstalk between GHS-R and insulin signaling in the pancreas.

However, our data differ from some previous reports that showed increased insulin secretion with GHS-R antagonist treatment [20] and *Ghsr*-knockin mice [25]. The discrepancies could be due to differences in the model systems and/or experimental conditions to study glucose-induced insulin secretion. In our study, islets were allowed to recover from the collagenase shock from islet isolation by incubating them overnight in RPMI1640, whereas Kurashina et al. used fresh islets for their study [25,92]. Fresh islets could be under acute stress conditions due to collagenase treatment and other purification steps. Acute and chronic stress have been previously shown to increase insulin secretion in isolated islets [93,94]. Further, we used 4.5–6.5-month-old mature adult mice, whereas others have used 2–2.5-month-old mice/rats, which are still undergoing developmental changes [20,25].

Our study also has several limitations. *First*, in our current study, we cannot eliminate the potential involvement of other factors and indirect effects of ghrelin on β-cells, that could well contribute to the GSIS phenotype. A) GHS-R1a is known to have a very high constitutive activity and can signal at 50% of its maximum capacity even in the absence of ghrelin [95]. B) Zigman et al. demonstrated that the blocking of GHS-R by the endogenous ligand LEAP2 or synthetic ligand [D-Lys3]-GHRP-6 increases pancreatic plasma polypeptide (PP) in both fed and fasted states [18]. PP is a well-known inhibitor of insulin secretion [96,97]. It could be informative to investigate the concentrations of PP in our *Ghsr*^−/−^ mice in the future. C) Recent studies using transcriptome profiling have demonstrated the GHS-R is highly expressed on somatostatin (SST) secreting δ-cells [14,22]. Ghrelin can stimulate SST release in isolated pancreatic islets, perfused pancreas, and healthy humans, thus ghrelin-induced SST can inhibit insulin secretion via SST receptors in β-cells [14,22,98,99,100,101]. Recently, ghrelin-induced SST stimulation was suggested to be mediated by GHS-R1a activation in δ-cells with the assistance of a GPCR accessory protein (Melanocortin Receptor Accessory Protein 2, MRAP2) [102]. We cannot eliminate the possibility of GHS-R1a independent ghrelin-mediated somatostatin release affecting GSIS in our studies. Ghrelin has been speculated to have GHS-R independent effects in other peripheral tissues [53,103,104,105]. It is possible that in our *Ghsr*^−/−^ mouse model, ghrelin is mediating its inhibitory effect on GSIS via yet unknown GPCRs in pancreatic islets. D) Other peptides, such as urocortin 3, which is highly expressed in β-cells, have been shown to inhibit somatostatin-mediated insulin secretion [106]. In the future, we may assess the urocortin 3 levels in our *Ghsr*^−/−^ mice. *Second*, the putative signaling regulators in our study need to be further verified. Our studies suggest the existence of a crosstalk between GHS-R and IR-IRS1/IRS2-PI3K/Akt signaling in pancreatic islets that contributes to attenuated GSIS. Though we see reduced gene expression of insulin signaling components in *Ghsr*^−/−^ islets, it is difficult to ascertain if it is a direct/causal effect of *Ghsr* on insulin signaling in β-cells. Further studies are needed to confirm the crosstalk between GHS-R and insulin signaling pathway, and verify if insulin signaling directly downregulates Pdx1 and contribute to GSIS. A very exciting recent report showed that ghrelin cell-selective insulin receptor-KO (GhIRKO) mice have reduced plasma insulin [107]; it would be interesting to investigate the insulin signaling in the β-cells of this mouse model. *Third*, we measured *Glut* 2 gene expression and glucose-metabolic intermediate G6P to assess glucose uptake into the cells. In the future, we should perform western blot or IHC to measure Glut 2 protein levels and provide more direct evidence of glucose imports in the β-cells.

Lastly, insulin resistance and impairment of GSIS in β-cells are hallmark features of diabetes. Our old *Ghsr*^−/−^ mice are protected from age-associated insulin resistance [27]. Here, we demonstrate that at a young age, these *Ghsr*^−/−^ mice exhibit reduced expression of insulin-signaling genes, lower GSIS, and high insulin sensitivity. It is possible that reduced GSIS and high insulin sensitivity at a young age protect the *Ghsr*^−/−^ mice from developing insulin resistance and T2D in aging [27].

In conclusion, our findings highlight that GHS-R is an important regulator of glucose-stimulated insulin secretion both in vivo and ex vivo, and suggest the existence of a crosstalk between GHS-R and insulin-signaling pathways in pancreatic islets. GHS-R likely regulates GSIS via the Akt-Pdx1-GLUT2 pathway in pancreatic β-cells; in-depth studies are warranted to validate the signaling pathway.
